# Long noncoding RNA XIST regulates brown preadipocytes differentiation and combats high-fat diet induced obesity by targeting C/EBPα

**DOI:** 10.1186/s10020-022-00434-3

**Published:** 2022-01-21

**Authors:** Chunyan Wu, Shu Fang, Huijian Zhang, Xiaoqiang Li, Yuejun Du, Yudan Zhang, Xiaochun Lin, Ling Wang, Xiaoqin Ma, Yaoming Xue, Meiping Guan

**Affiliations:** 1 Department of Endocrinology and Metabolism, Nanfang Hospital, Southern Medical University, Guangzhou, 510515 China; 2 Department of Endocrinology and Metabolism, The Third Affiliated Hospital of Southern Medical University, Guangzhou, 510515 China; 3 Department of Urology, Nanfang Hospital, Southern Medical University, Guangzhou, 510515 China; 4 Department of Plastic Surgery, Nanfang Hospital, Southern Medical University, Guangzhou, 510515 China

**Keywords:** lncRNA, XIST, Brown preadipocyte, Adipose tissue, Obesity

## Abstract

**Background:**

Activation of brown adipose tissue (BAT) increases energy expenditure, which makes it an attractive therapeutic strategy for obesity. LncRNAs play an important role in adipocyte differentiation and regulation. Here we assessed the effect of lncRNA XIST on brown preadipocytes differentiation and metabolic regulation.

**Methods:**

XIST expression levels were detected in human perirenal (peri-N) and subcutaneous adipose tissues (sub-Q), brown preadipocytes and 3T3-L1 preadipocytes. XIST overexpression and knockdown experiments were performed in brown preadipocytes. XIST overexpression mouse model was established by plasmid injection through tail vein.

**Results:**

In human adipose tissues, XIST expression was significantly higher in female than in male individuals. In vitro, XIST expression was significantly up-regulated during brown adipocyte differentiation. XIST knockdown inhibited differentiation of brown preadipocytes, while overexpression of XIST promotes brown preadipocytes to fully differentiation. RNA Binding Protein Immunoprecipitation (RIP) experiment revealed that XIST could directly bind to C/EBPα. In vivo, XIST overexpression prevents high-fat diet induced obesity and improves metabolic dysorder in male mice.

**Conclusion:**

Our results suggest that XIST combats obesity through BAT activation at least partly by combination with transcription factor C/EBPα.

**Supplementary Information:**

The online version contains supplementary material available at 10.1186/s10020-022-00434-3.

## Introduction

Adipose tissue plays an important role in metabolic homeostasis. Obesity is characterized by excessive accumulation of adipose tissue, leading to an increase risk of metabolic diseases (Blüher 2019). In humans, adipose tissue distribution is different between males and females. Men accumulate more visceral fat tissue, while premenopausal women accumulate more subcutaneous fat tissue (Palmer and Clegg 2015). And these fat distribution pattern leads to typical women “Pear-shaped” obesity and male “apple-shaped” obesity (Gesta et al. 2007).

Previous studies suggest that lncRNA plays an important role in the differentiation of adipose tissue (Tran et al. 2020; Xiao 2021). X inactive-specific transcript (XIST) lncRNA is the critical regulator in X-chromosome inactivation (Payer and Lee 2008), which play an important role in X–linked gene expression balance between sexes. XIST is also involved in cell differentiation, proliferation, X-chromosome inactivation, tumorigenesis and immunity (Yildirim et al. 2013; Wang et al. 2016; Wang et al. 2021; Wang et al. 2020; Salama et al. 2019). Clinically, women are more likely to suffer from certain diseases than man are, including autoimmune diseases and Cushing syndrome. Recent research (Vasanthakumar et al. 2020) found that regulatory T (Treg) cells exhibit sexual dimorphism in visceral adipose tissue (VAT), more Treg cells are present in male mouse VAT compared with female VAT. Treg cells from VAT were significantly different in phenotype and transcriptional landscape between male and female. Previous studies from our group (Wu et al. 2019) showed that patients with cortisol-producing adenoma have obvious oxidative stress and inflammation. Further sequence studies and preliminary research revealed that the expression of lncRNA XIST in subcutaneous fat in female cortisol-producing adenoma patients was significantly higher than that of female normal controls, and the expression of lncRNA XIST in female adipose tissue was significantly higher than that in males. Previous study (Shinozaki et al. 2007) has shown that XIST was expressed in female ob/ob mouse adipose tissue. However, the role of XIST in adipocyte differentiation and function is still unclear. In the present study, we further investigate the impact of XIST on adipose tissue, and explore its effect on brown preadipocytes differentiation and possible mechanism.

## Methods

### Human adipose tissue

Perirenal adipose tissues (peri-N) and subcutaneous adipose tissues (sub-Q) were obtained from 5 female patients (Female CON) and 6 male patients (Male CON) undergoing laparoscopic nephrolithotomy or ureterolithotomy. All subjects involved in this study have signed informed consent. The study was approved by the Ethics Committee of Nanfang Hospital.

### Cell culture

Mouse 3T3-L1 preadipocytes were purchased from type culture collection of the Chinese Academy of Sciences. Mouse brown preadipocytes were established as previously described (Fasshauer et al. 2001; Klein et al. 1999). 3T3-L1 preadipocytes and brown preadipocytes were induced to differentiate as previously described (Zhang et al. 2015). 3T3-L1 and brown preadipocytes postconfluent 2 days designated as day 0. For XIST knockdown, 50 nM XIST Smart Silencer (RiboBio, China) was transfected into brown preadipocytes using Lipofectamine 3000 (Invitrogen, USA). For XIST overexpression, brown preadipocytes were transfected with XIST overexpression plasmid (mouse pCMV-XIST, Addgene) using Lipofectamine 3000 according to the operation manual. 3T3-L1 preadipocytes and brown preadipocytes were harvested at the indicated time points.

### Quantitative real-time PCR and western blot

Total RNA from human adipose tissues, mouse adipose tissues and cells were extracted using Trizol reagent (TAKARA). cDNA was synthesized using a PrimeScript RT Reagent Kit (TAKARA). Quantitative Real-Time PCR was performed on a Roche LightCycler 480 Real-Time PCR system. Mouse Arbp was used as internal control in cells and mouse adipose tissues, and human 18S was chosen as normalization control in human adipose tissues. Protein expressions were detected by western blot as previously reported (Cai et al. 2019). The primary antibodies were specific for Sterol Regulator Elementing Binding Protein 1c (SREBP1C) (Abclonal), adiponectin (CST), peroxisome proliferator-activated receptor γ (PPARγ) (CST), CCAAT-enhancer-binding protein α (C/EBPα) (Abcam), uncoupling protein 1 (UCP1) (Santa), PPARγ coactivator-1α (PGC1α) (Santa), Wnt Family Member 10B (Wnt10B) (Abclonal), SMAD Family Member 2 (SMAD2) (CST), phosphor-SMAD2 (CST), and β-ACTIN server as loading control.

### Nuclear and cytoplasmic extraction and RNA-binding protein immunoprecipitation (RIP)

Nuclear and cytoplasmic extraction of mouse brown preadipocytes and mature brown preadipocytes was prepared with NE-PER Nuclear and Cytoplasmic Extraction Reagents (Thermo Scientific) according to manufacturer’s protocol. RIP assays were performed using RNA-Binding Protein Immunoprecipitation Kit (Millopore) according to the manufacturer’s recommendations. 5 μg Rabbit anti-C/EBPα (Abcam) and IgG control antibodies were used to perform RIP assay. The enrichment of XIST was detected by real-time reverse transcription PCR (qRT-PCR).

### Animals

The Animal Experimental Committee at Nanfang Hospital approved all mouse procedures. The 6 weeks old male C57BL/6 J mice were obtained from Guangdong Medical Laboratory Animal Center (Guangzhou, China). Male C57BL/6 J mice were randomly divided into four groups and fed either chow diet (CD) or high-fat diet (HFD, 60% kcal from fat). For in vivo XIST overexpression, male 8 weeks old C57BL/6 J mice were injected with pCMV-XIST plasmid or control plasmid diluted with PBS via tail vein every 4 weeks (Kong et al. 2015). Intraperitoneal glucose tolerance test (IPGTT) and intraperitoneal insulin tolerance test (IPITT) were conducted at week 20 and week 21 of age respectively. The IPGTT and IPITT were carried out as previously describe (Cao et al. 2013). At week 22, blood sample and adipose tissues were harvested.

### Serum lipid determination

Serum samples were analyzed for triglycerides (TG), total cholesterol, low-density lipoprotein cholesterol (LDL-C) and high-density lipoprotein cholesterol (HDL-C) using commercially available kits (Nanjing Jiancheng Bioengineering Institute) according to the manufacturer’s instructions.

### Histology

Mice adipose tissues were fixed in formaldehyde and embedded in paraffin. Paraffin sections of BAT and sub-Q isolated from male mice were stained using hematoxylin–eosin as previously described (Xu et al. 2017).

### Statistical analysis

Statistical significance was determined with the Student’s test for normally distributed data, and results were presented as mean ± standard error.

## Results

### Expression of XIST in human adipose tissues and mice adipocytes

**Fig. 1:**
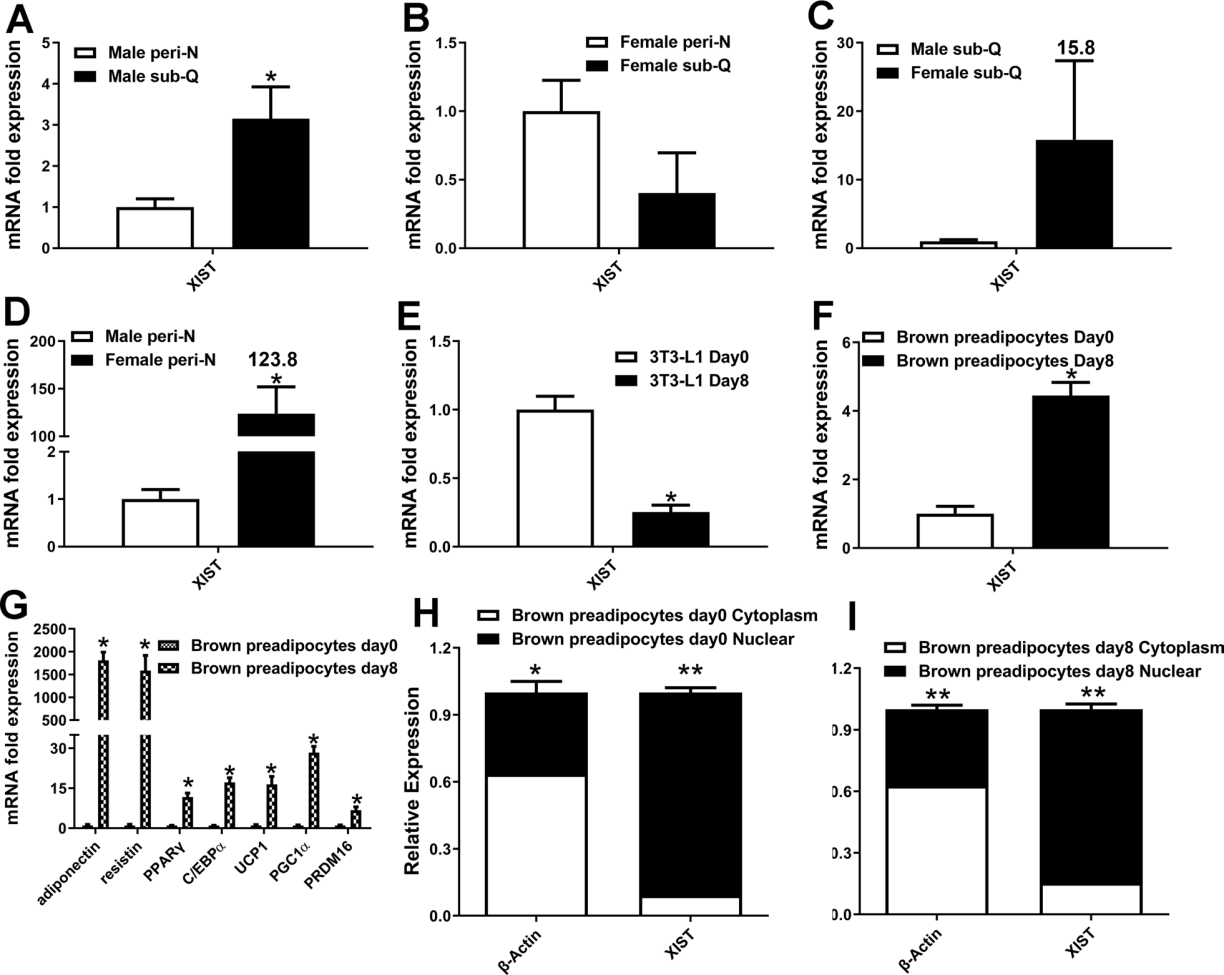
XIST expression in human adipose tissues and preadipocytes. **A**–**D** qRT-PCR analyses of XIST expression in human perirenal and subcutaneous adipose tissues. **E** mRNA level of XIST in white (3T3-L1) preadipocytes. **F**–**G** qRT-PCR analyses of XIST and indicated genes in brown preadipocytes. **H**–**I** Subcellular localization of brown preadipocytes. Male: male patients, n = 6; Female: female patients, n = 5. peri-N: perirenal adipose tissue; sub-Q: subcutaneous adipose tissue. Day 0: undifferentiated adipocytes; day 8: differentiated adipocytes. *, p < 0.05; **, p < 0.01. Data are represented as mean ± SEM

To explore whether lncRNA XIST is involved in the regulation of adipose tissue function, XIST was measured in adipose tissues. There was a different deposit-specific pattern in XIST expression between male and female. XIST expression was significantly higher in male subcutaneous adipose tissues than perirenal adipose tissues (Fig. 1A), which was inverse in female although with no significance. In perirenal adipose tissue XIST expression was significantly higher in female than in male individuals (×123.8). In sub-Q although there is no statistical difference, as shown in Fig. 1, XIST expression was obviously higher in female than in male (×15.8). To investigate the potential function of XIST in adipocytes, we examined XIST expression in preadipocytes and mature adipocytes. XIST expression was significantly depressed during white adipocyte differentiation, while it was significantly up-regulated during brown adipocyte differentiation. XIST was expressed predominantly in the nucleus in both brown preadipocytes and mature brown preadipocytes. Owing to the high expression levels of XIST in differentiated brown preadipocytes, we assumed that XIST might have an important role in brown adipogenesis.

### Overexpression of XIST stimulates the differentiation of brown preadipocytes

**Fig. 2:**
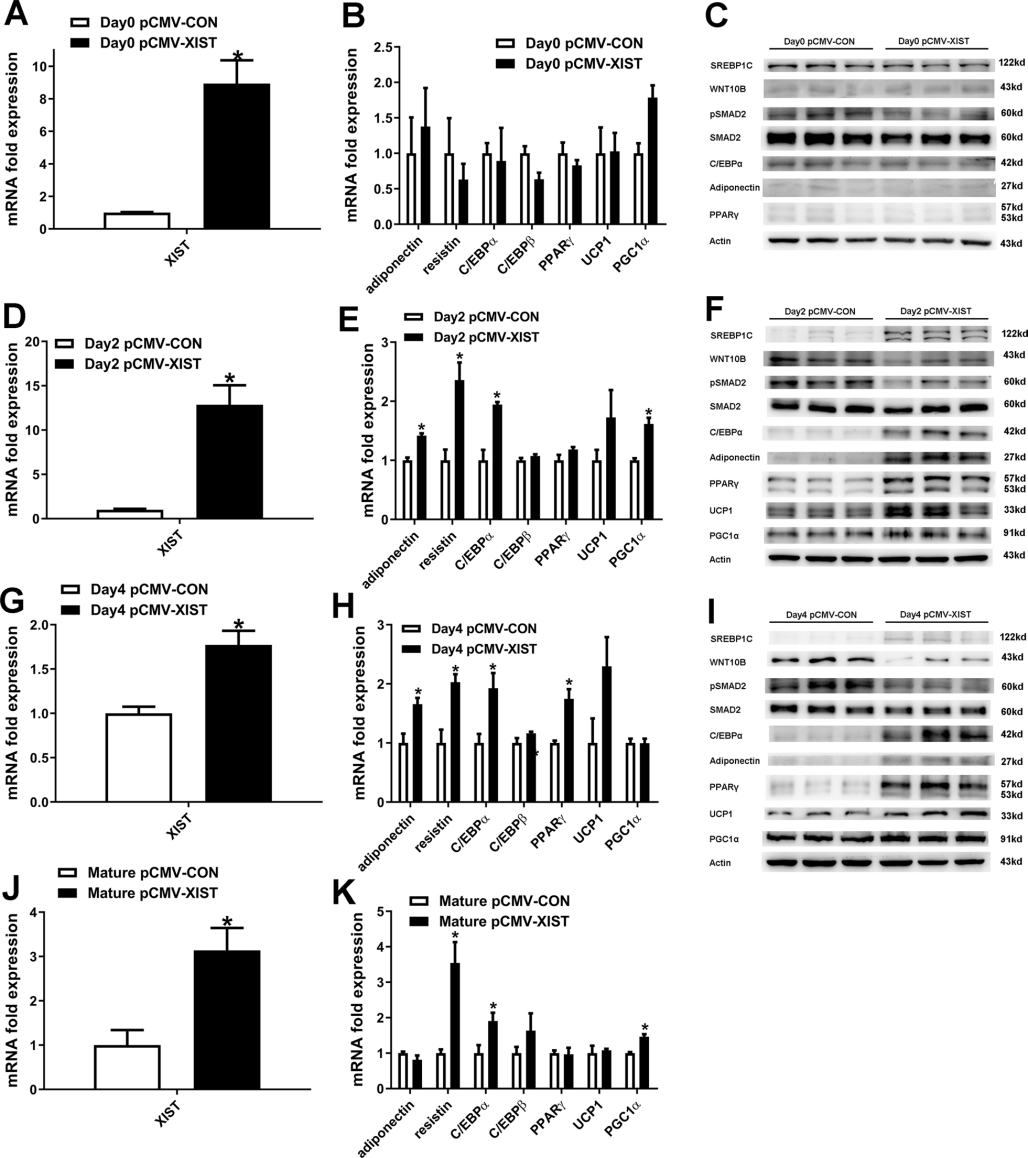
Overexpression of XIST promotes brown preadipocyte differentiation. **A**–**B**, **D**–**E**, **G**–**H**, qRT-PCR analysis of XIST and indicated genes during the differentiation of brown preadipocytes transfected with control or XIST overexpression plasmid. **C**, **F**, **I** Western blot analysis of the expression of indicated proteins during the differentiation of brown preadipocytes transfected with control or XIST overexpression plasmid. **J**–**K** qPCR analysis of XIST and indicated genes in differentiated brown preadipocytes transfected with control or XIST overexpression plasmid. *, p < 0.05. Data are represented as mean ± SEM

To determine the effect of XIST overexpression on brown preadipocytes differentiation, adipokines and differentiation markers were examined during the differentiation of brown preadipocytes. XIST overexpression was comfirmed by qRT-PCR (Fig. 2A, D, G, J). On day 0, overexpression of XIST had no effect on adipokines and differentiation markers (Fig. 2B, C). On day 2 and day 4 after induction of differentiation, XIST overexpression significantly increased mRNA expressions of adiponectin, resistin and C/EBPα, and protein expressions of SREBP1C, adiponectin, C/EBPα, PPARγ and UCP1 (Fig. 2E, F, H, I). Furthermore, overexpression of XIST led to a decreased expression of SMAD2 phosphorylation and WNT10B protein expression. We also investigated whether XIST overexpression affected mature brown preadipocytes. Overexpression of XIST increased resistin, C/EBPα and PGC1α mRNA expression in mature brown preadipocytes (Fig. 2K). These results indicated that overexpression of XIST enhanced brown preadipocytes differentiation.

### Effects of XIST knockdown on brown preadipocytes

**Fig. 3:**
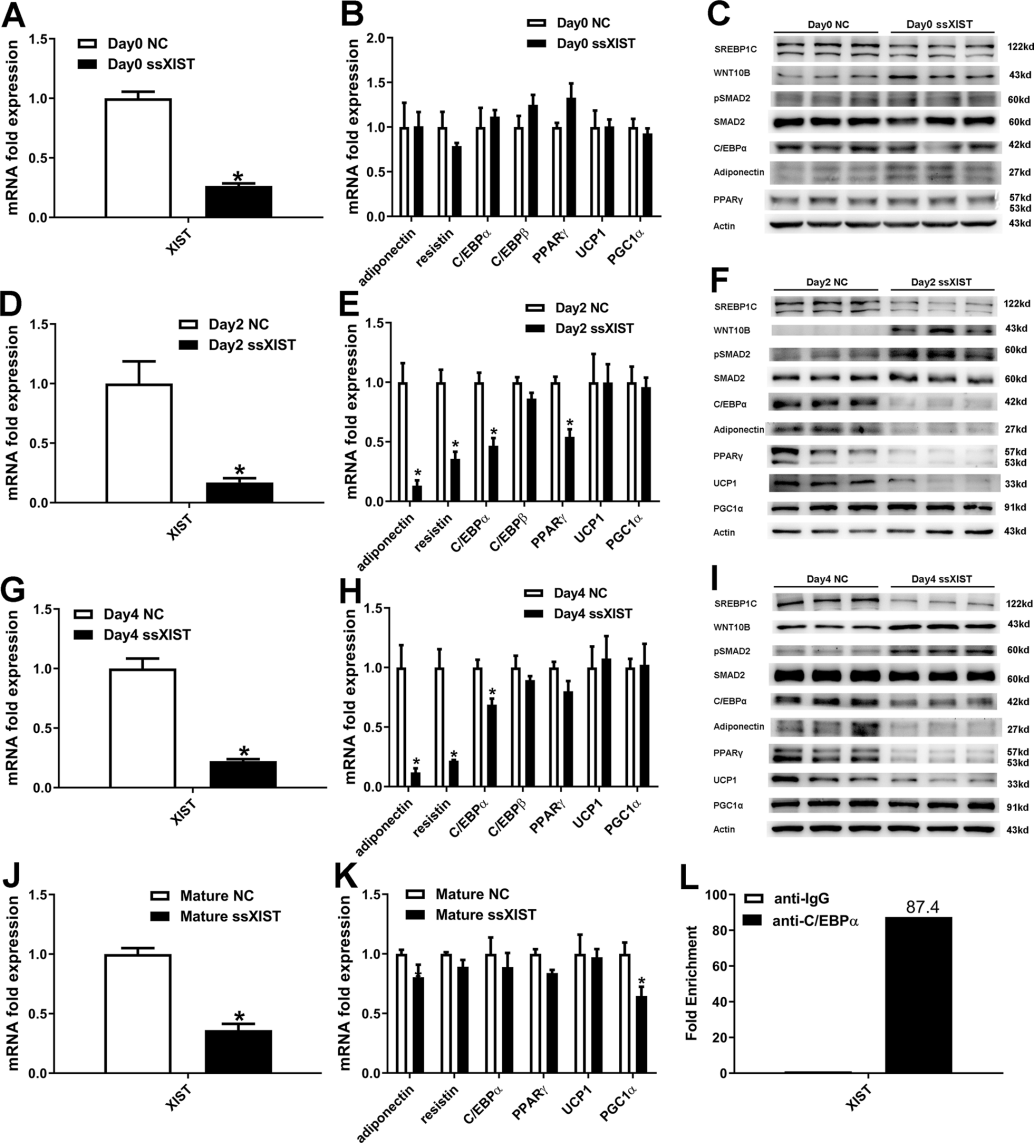
Silencing of XIST inhibits brown preadipocyte differentiation. **A**–**B**, **D**–**E**, **G**–**H** qRT-PCR analysis of XIST and indicated genes during the differentiation of brown preadipocytes transfected with control or XIST smart silencer. **C**, **F**, **I** Western blot analysis of the expression of indicated proteins during the differentiation of brown preadipocytes transfected with control or XIST smart silencer. **J**–**K** qRT-PCR analysis of XIST and indicated genes in differentiated brown preadipocytes transfected with control or XIST smart silencer. **L** RNA-binding protein immunoprecipitation (RIP) assay showing the interaction between XIST and C/EBPα. *, p < 0.05. Data are represented as mean ± SEM

To further explored the role of XIST in brown preadipocyte differentiation, we transfected brown preadipocytes with XIST smart silencer or negative control (NC). XIST knockdown was validated by qRT-PCR. On day 0, knockdown of XIST had no effect on adipokines and differentiation markers in brown preadipocytes. On day 2 and day 4 after induction of differentiation, we found that knockdown of XIST decreased adiponectin, resistin and C/EBPα mRNA expression. We also found that knockdown of XIST led to a decreased expression of SREBP1C, adiponectin, C/EBPα, PPARγ and UCP1 protein expression, but increased SMAD2 phosphorylation and WNT10B protein expression. As shown in Fig. 3K, XIST knockdown decreased PGC1α mRNA expression but not others, thereby suggesting that knockdown of XIST had little effect on differentiated brown preadipocytes. RIP experiment confirmed that XIST could directly bind to C/EBPα (Fig. 3L). Together, our results indicated that XIST involved in brown preadipocytes differentiation at least partly through combination with C/EBPα.

### Overexpression of XIST prevents metabolic dysorders induced by HFD induced male mice

**Fig. 4:**
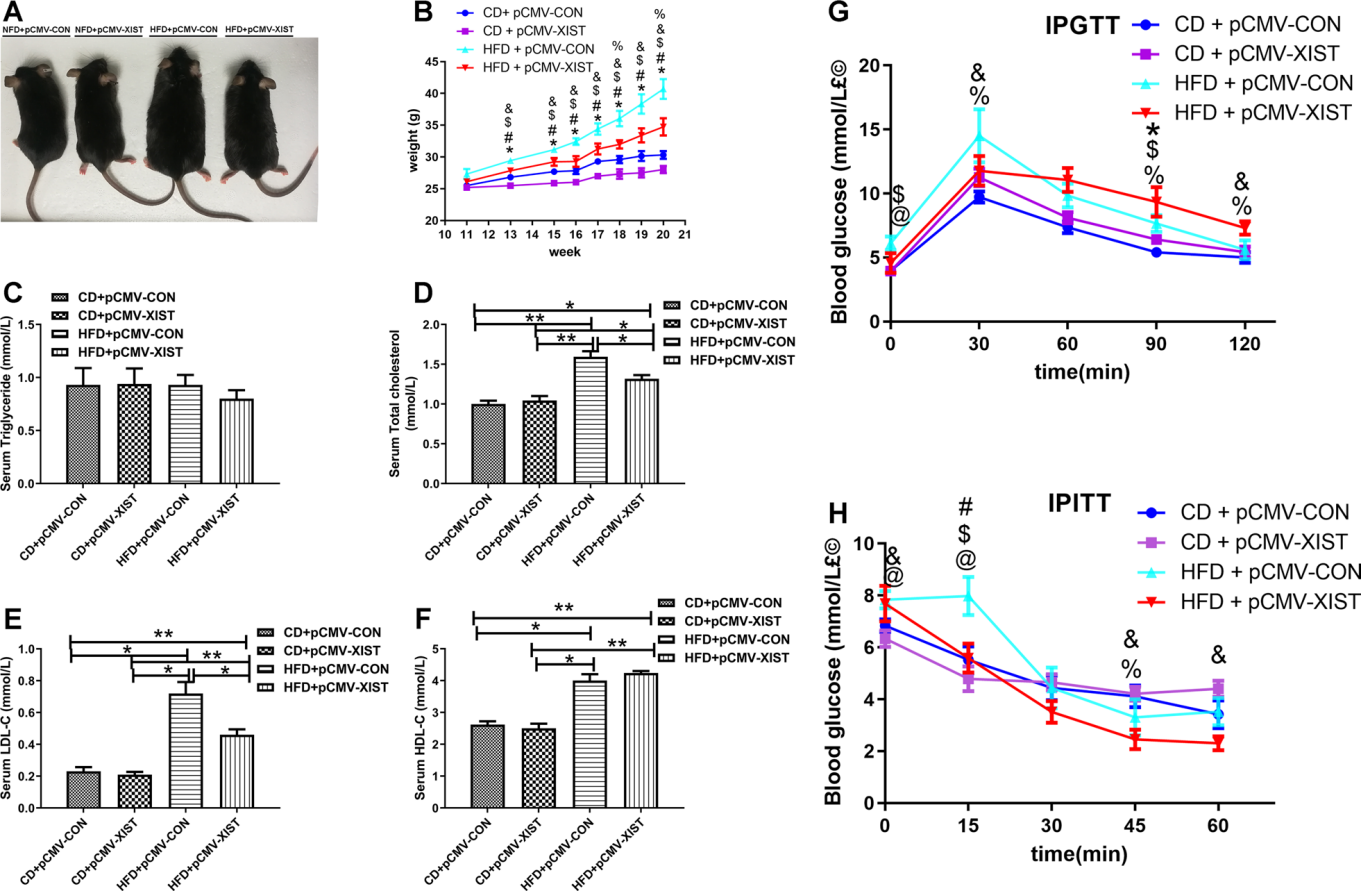
XIST overexpression attenuates HFD-induced obesity in vivo. **A** Representative photograph of male mouse whole body images between groups. **B** Body weight of male mice between groups. CD, chow diet. HFD, high fat diet. *CD + pCMV-CON (n = 6) vs CD + pCMV-XIST (n = 6), p < 0.05; # HFD + pCMV-CON (n = 6) vs HFD + pCMV-XIST (n = 6), p < 0.05; $ CD + pCMV-CON vs HFD + pCMV-CON, p < 0.05; and CD + pCMV-XIST vs HFD + pCMV-XIST, p < 0.05; % CD + pCMV-CON vs HFD + pCMV-XIST, p < 0.05. **G**–**H** Insulin tolerance test and glucose tolerance test of male mice between groups. **C**–**F** Serum triglyceride, cholesterol, LDL-C and HDL-C levels in male mice between groups. *, p < 0.05. Data are represented as mean ± SEM

**Fig. 5:**
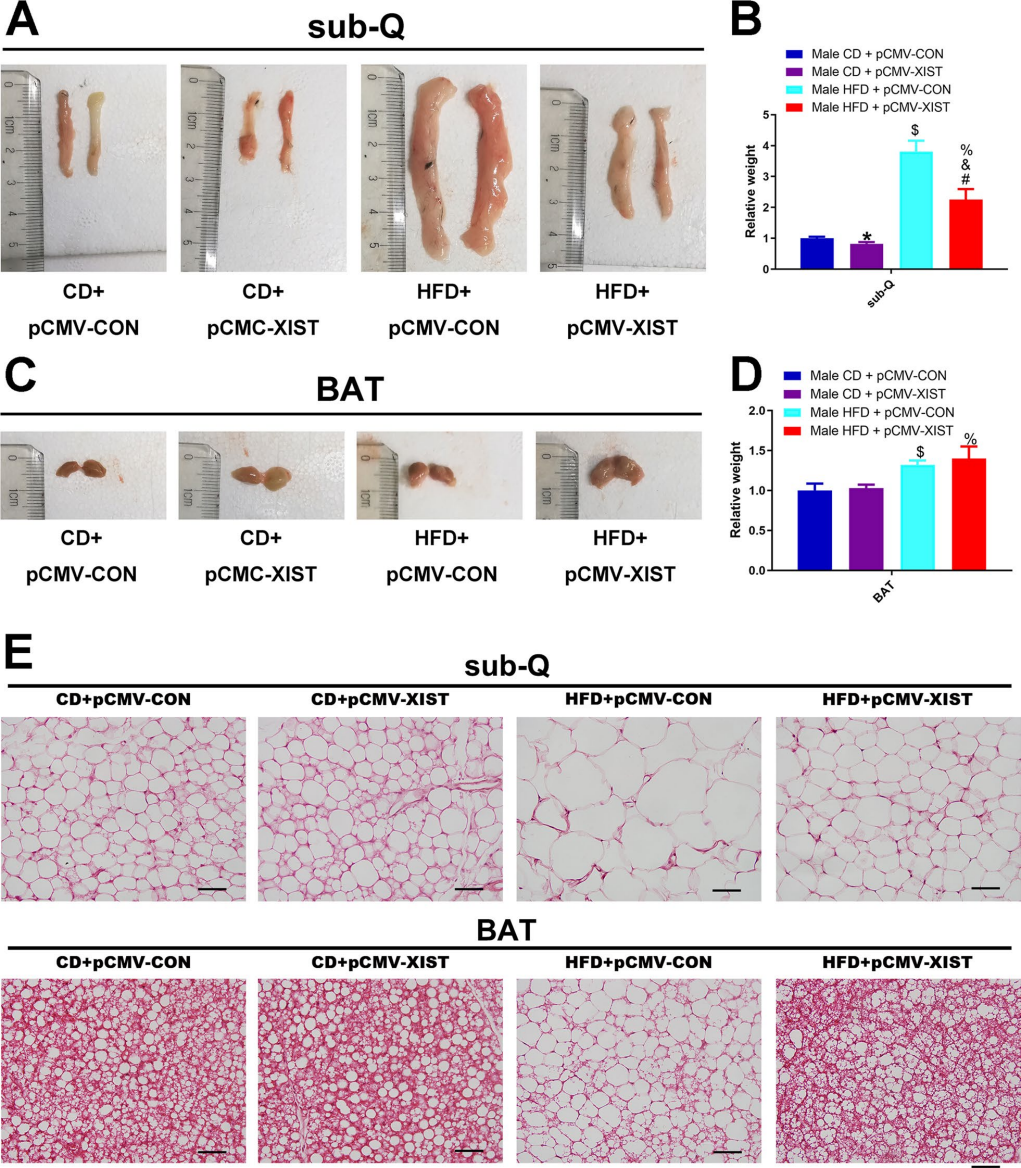
XIST overexpression attenuates adipose tissue histological changes in HFD-induced mice. **A**–**D** Tissue weight in male mice between groups. * vs CD + pCMV-CON, p < 0.05; # vs HFD + pCMV-CON, p < 0.05; $ vs CD + pCMV-CON, p < 0.05; & vs CD + pCMV-XIST p < 0.05; % vs CD + pCMV-CON, p < 0.05. **E** Representative HE stained subcutaneous and brown adipose tissue of male mice between groups. Scale bar, 50 μm

**Fig. 6:**
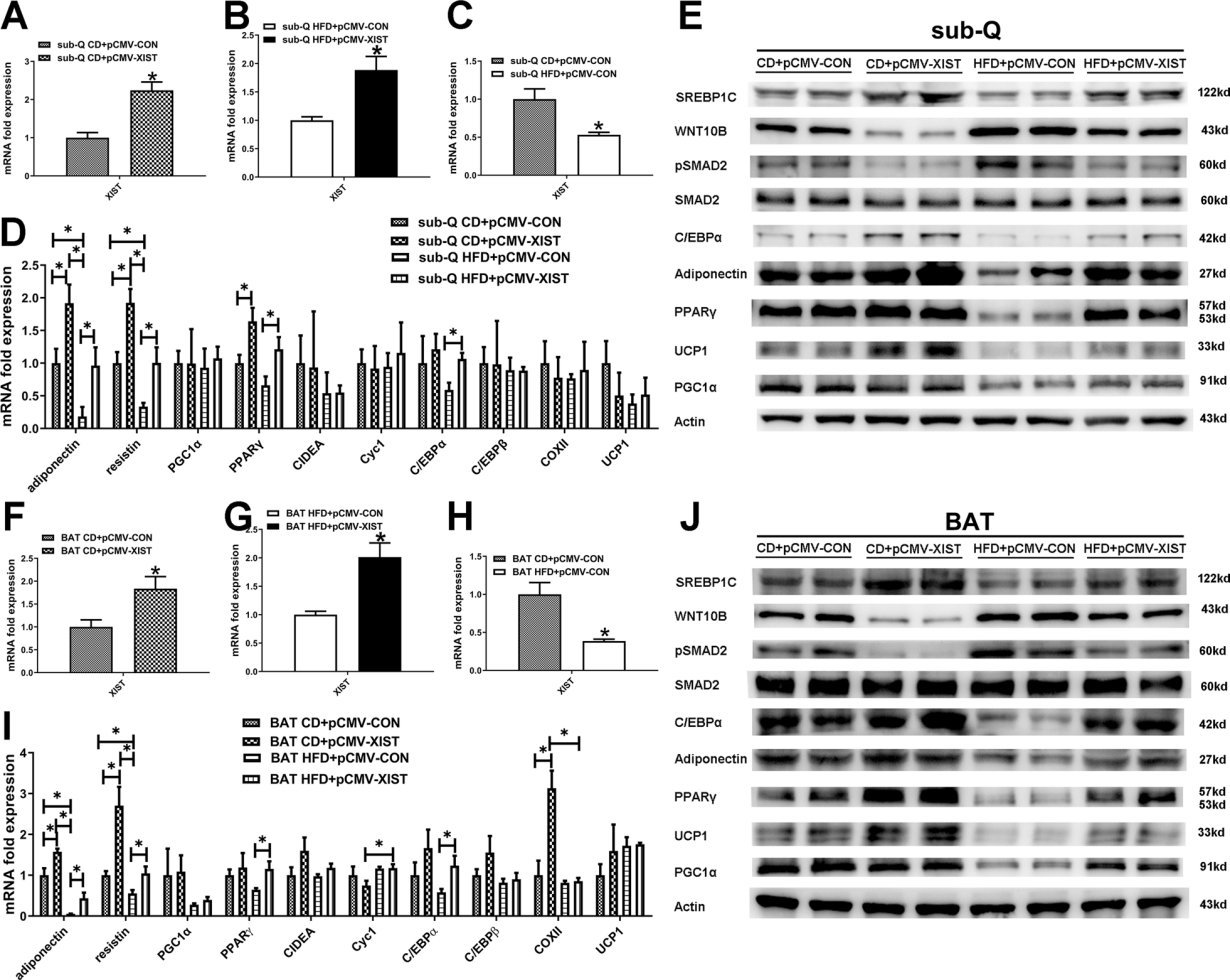
XIST overexpression effect on metabolism-related gene expression of adipose tissues in HFD-induced mice. **A**–**D**, **F**–**I** qRT-PCR analysis of XIST and indicated gene expression in sub-Q and BAT from male mice after intervention. *, p < 0.05. **E**, **J** Western blot analysis of indicated protein expression in sub-Q and BAT from male mice after intervention. CD, chow diet; HFD, high-fat diet; pCMV-CON, tail vein injection of control plasmid; pCMV-XIST, tail vein injection of XIST overexpression plasmid; BAT, brown adipose tissue; sub-Q, subcutaneous adipose tissue; LDL-C, low-density lipoprotein cholesterol; HDL-C, high-density lipoprotein cholesterol. Data are represented as mean ± SEM

To further explore the role of XIST in HFD induced obesity, we overexpressed XIST by plasmid injection via tale vein in male mice. Mice fed with HFD exhibited an increased body weight gain compared to chow diet (CD) fed mice (Fig. 4A, B). The weight gain in HFD mice was abolished by overexpression of XIST (Fig. 4A, B). To investigate the effects of XIST on glucose homeostasis, we performed IPGTT and IPITT. As shown in Fig. 4G, H HFD and XIST overexpression had little effect on IPGTT and IPITT. We also found that HFD and XIST overexpression had no effect on serum triglyceride (Fig. 4C). Serum total cholesterol, LDL-C and HDL-C were increased in HFD mice compared to CD control (Fig. 4D–F). Overexpression of XIST resulted in a decreased serum total cholesterol and LDL-C in HFD + pCMV-XIST mice compared to HFD + pCMV-CON group (Fig. 4D, E). HFD-induced mice displayed significantly increased weights of BAT and sub-Q than CD control, whereas overexpression of XIST alleviated sub-Q weight increased induced by HFD (Fig. 5A–D). BAT weight was slightly but not significantly increased in HFD + pCMV-XIST mice (Fig. 5C, D). To further reveal the mechanism of adipose tissue weight change in HFD + pCMV-XIST mice, we performed H&E staining. Histological analyses showed that adipocytes were larger in both sub-Q and BAT of HFD mice than CD control (Fig. 5E). Besides, XIST overexpression in HFD mice resulted in decreased size of adipocytes in both BAT and sub-Q compared to the HFD + pCMV-CON mice, indicating that XIST overexpression could suppress HFD induced adipocyte hypertrophy (Fig. 5E). Overexpression of XIST was confirmed in sub-Q and BAT by qRT-PCR (Fig. 6A, B, F, G). We also found that XIST expression decreased significantly in sub-Q and BAT of HFD male mice compared with CD control (Fig. 6C, H). It was shown that the mRNA expressions of adiponectin and resistin were significantly down-regulated in sub-Q and BAT of HFD + pCMV-CON mice compared with CD control, while overexpression of XIST prevented the downregulation of above genes in sub-Q and BAT induced by HFD (Fig. 6D, I). The protein expressions of SREBP1C, adiponectin, C/EBPα, PPARγand UCP1 were lower in sub-Q and BAT from HFD + pCMV-CON mice compared to CD control, while overexpression of XIST in HFD mice restored above proteins expression to that observed in CD mice (Fig. 6E, J). We also found that HFD increased WNT10B protein and SMAD2 phosphorylation in BAT and sub-Q compared with CD control, which was inhibited by XIST overexpression (Fig. 6E, J).

## Discussion

The incidence of obesity and related metabolic comorbidities is increasing worldwide. However, there is few safe and efficacious medicines for treating obesity (Müller et al. 2018). In this study, we found that XIST is significantly increased during brown preadipocytes differentiation, but decreased during differentiation of 3T3-L1 preadipocytes. XIST overexpression promotes brown preadipocytes differentiation whereas knockdown of XIST inhibits it. In vivo experiments confirmed that XIST overexpression exhibited protection from HFD induced obesity and improved adipose tissue function.

Brown adipose tissue is characteristic by non-shivering thermogenesis which plays an important role in energy consumption. It is known that lncRNA can regulate pathophysiological process in several ways (Marchese et al. 2017). Accumulating evidence suggests that lncRNA involved in adipocyte differentiation and regulation. Zhao et al. (2014) employed globally profiled lncRNA gene expression and identified lncRNA Blnc1 promoting brown adipocyte differentiation. Similarly, lnc-BATE1 is another brown adipose tissue-specific lncRNA required for establishment and maintenance of brown adipose tissue identity (Alvarez-Dominguez et al. 2015).

XIST plays a key role in X-linked gene expression balance and participates in multiple pathophysiological process. Our data shows that XIST expression is significantly higher in female than in male adipose tissue. Recent studies showed that there were sex differences in brown adipose tissue activity (Kaikaew et al. 2021). Compared with males, female rodents have larger brown adipose tissue mass and higher prevalence of active brown adipose tissue (Kim et al. 2016). Female rats have higher UCP1 content and higher dense mitochondria in brown adipose tissue than male rats (Rodriguez-Cuenca et al. 2002). In healthy women (Link and Reue 2017), intraperitoneal adipocytes are smaller than subcutaneous adipocytes, while in men, the adipocytes size in these two regions are similar. Our results shown that the expression of XIST is significantly increased during brown preadipocytes differentiation, but markedly decreased during differentiation of 3T3-L1 preadipocytes. Besides, the expression of key adipokines, including UCP1, C/EBPα, PPARγ and adiponectin could be upregulated by XIST overexpression. UCP1 is a protein expressed in mitochondrial inner membrane, which is mainly expressed in brown adipose tissue (Ricquier and Bouillaud 2000). Nonshivering thermogenesis is mediated by UCP1 in BAT. PPARγ and C/EBPα are important transcription factors that are essential for adipogenesis (Wu et al. 1999). Adiponectin has previously reported (Fu et al. 2005) to promote differentiation in 3T3-L1 preadipocytes. In vitro studies it has shown that resistin affects lipid metabolism during adipocyte differentiation. Ikeda et al. reported (Ikeda et al. 2013) that resistin knockdown significantly decreased lipid contents in 3T3-L1 preadipocytes during differentiation. Previous study reported (Kim et al. 1998) that SREBP1C promoted adipogenic in vitro. Although SREBP1 knockout mice (Shimano et al. 1997) did not show significant effects on adipose tissue development.

In addition, our results indicated that overexpression of XIST decreased SMAD2 phosphorylation and WNT10B protein expression in brown preadipocytes during differentiation. Lee et al. (2019) reported that adipose stem cells (ASCS) from human omental adipose tissue showed lower adipogenesis compared with abdominal fat, which associated with increased levels of TGFβ ligands that activate SMAD2 and suppress adipogenesis. Previous report (Zhu et al. 2018) indicated that SnoN promoted adipogenic differentiation by repressed activin/SMAD2 signaling. WNT10B has been previously shown (Ross et al. 2000) to be an effective adipogenesis inhibitor, and its expression level decreased during adipocyte differentiation. In vitro study (Ross et al. 2000) confirmed that ectopic expression of WNT10B significantly inhibited adipocyte differentiation. FABP4-WNT10B transgenic mice shown (Longo et al. 2004) that expression of WNT10B in adipose impairs adipose tissue development.

XIST is primarily localized in nucleus and RNA-binding immunoprecipitation confirmed the binding of XIST with C/EBPα. It has been confirmed (Hamm et al. 2001) both in vitro and in vivo experiments that C/EBPα was one of important regulators of adipocyte differentiation. C/EBPα and PPARγ cooperatively regulate adipocyte biology (Lefterova et al. 2008). Previous study reported (Freytag et al. 1994) that ectopic expression of C/EBPα effectively promoted adipogenic program of a variety of mouse fibroblasts. Thus, in this study, XIST involves in brown preadipocytes differentiation partly by combination with C/EBPα. Since the background expression level of XIST in male mice is lower than female mice, and whether overexpression of XIST in male mice can improve adipose tissue metabolism is still unknown, therefore, we further selected male mice for in vivo experiments. In vivo overexpression of lncRNA XIST can resist HFD-induced weight gain and improve adipose tissue function. Our research suggests that there are still much to explore in obesity and its related adipose differentiation and metabolism between the genders, which is far more than the difference of sex hormones.

Collectively, our results demonstrate that XIST plays an important role in brown preadipocytes differentiation at least partly through combinating with transcription factor C/EBPα. LncRNA XIST could be a potential target for combatting obesity by activating BAT.

## Supplementary Information

**Additional file 1: Table S1. **The sequences of primers used for RT-PCR.

## Data Availability

All data generated or analysed during this study are included in this published article and its Additional file 1.

## Abbreviations

XIST: X inactive-specific transcript
BAT: Brown adipose tissue
peri-N: Perirenal adipose tissues
sub-Q: Subcutaneous adipose tissues
VAT: Visceral adipose tissue
RIP: RNA Binding Protein Immunoprecipitation
Treg: Regulatory T
SREBP1C: Sterol Regulator Elementing Binding Protein 1c
PPARγ: Peroxisome proliferator-activated receptor γ
C/EBPα: CCAAT-enhancer-binding protein α
UCP1: Uncoupling protein 1
PGC1α: PPARγ coactivator-1α
Wnt10B: Wnt Family Member 10B
SMAD2: SMAD Family Member 2
qRT-PCR: Real-time reverse transcription PCR
IPGTT: Intraperitoneal glucose tolerance test
IPITT: Intraperitoneal insulin tolerance test
TG: Triglycerides
LDL-C: Low-density lipoprotein cholesterol
HDL-C: High-density lipoprotein cholesterol
CD: Chow diet
HFD: High fat diet
