# Supplementary material for: Long noncoding RNA XIST regulates brown preadipocytes differentiation and combats high-fat diet induced obesity by targeting C/EBPα
Source: Mol Med. 2022 Jan 21;28:6. doi: 10.1186/s10020-022-00434-3 (PMC8781062; doi:10.1186/s10020-022-00434-3)
Supplement: Supplementary file 1 — Additional file 1: Table S1. The sequences of primers used for RT-PCR. [file 10020_2022_434_MOESM1_ESM.docx]

**Table S1**. The sequences of primers used for RT-PCR.

| **human** | | **Primer sequences** | **GenBank**  **Accession number** | **product length** | **annealing temperature** |
| --- | --- | --- | --- | --- | --- |
| 18S | Forward | TCAACTTTCGATGGTAGTCGCCGT | NM_022551 | 108bp | 60℃ |
|  | Reverse | TCCTTGGATGTGGTAGCCGTTTCT |  |  |  |
| XIST | Forward | ACGCTGCATGTGTCCTTAGTAGTC | NR_001564 | 102bp | 60℃ |
|  | Reverse | ATTTGGAGCCTCTTATAGCTGTTTG |  |  |  |
| **mouse** | | **Primer sequences** | **GenBank**  **Accession number** | **product length** | **annealing temperature** |
| Arbp | Forward | TTTGGGCATCACCACGAAAA | NM_007475 | 239bp | 60℃ |
|  | Reverse | GGACACCCTCCAGAAAGCGA |  |  |  |
| β-ACTIN | Forward | GTCCACCCCGGGGAAGGTGA | NM_007393 | 126bp | 60℃ |
|  | Reverse | AGGCCTCAGACCTGGGCCATT |  |  |  |
| XIST | Forward | ATCTAAGACAAAATACATCATTCCG | NR_001570 | 250bp | 60℃ |
|  | Reverse | CTTGGACTTAGCTCAGGTTTTGTGTC |  |  |  |
| adiponectin | Forward | TGTTCCTCTTAATCCTGCCCA | NM_009605 | 104bp | 60℃ |
|  | Reverse | CCAACCTGCACAAGTTCCCTT |  |  |  |
| resistin | Forward | ACAAGACTTCAACTCCCTGTTTC | NM_022984 | 161bp | 60℃ |
|  | Reverse | TTTCTTCACGAATGTCCCACG |  |  |  |
| PPARγ | Forward | TCAGCTCTGTGGACCTCTCC | NM_001127330 | 350bp | 60℃ |
|  | Reverse | ACCCTTGCATCCTTCACAAG |  |  |  |
| UCP1 | Forward | AGGCTTCCAGTACCATTAGGT | NM_009463 | 133bp | 60℃ |
|  | Reverse | CTGAGTGAGGCAAAGCTGATTT |  |  |  |
| PGC1α | Forward | CCCTGCCATTGTTAAGACC | NM_008904 | 161bp | 60℃ |
|  | Reverse | TGCTGCTGTTCCTGTTTTC |  |  |  |
| C/EBPα | Forward | CAAGAACAGCAACGAGTACCG | NM_001287514 | 124bp | 60℃ |
|  | Reverse | GTCACTGGTCAACTCCAGCAC |  |  |  |
| C/EBPβ | Forward | GGACTTGATGCAATCCGGA | NM_001287738 | 119bp | 60℃ |
|  | Reverse | AACCCCGCAGGAACATCTTTA |  |  |  |
| CIDEA | Forward | ATCACAACTGGCCTGGTTACG | NM_007702 | 136bp | 60℃ |
|  | Reverse | TACTACCCGGTGTCCATTTCT |  |  |  |
| PRDM16 | Forward | CAGCACGGTGAAGCCATTC | NM_027504 | 87bp | 60℃ |
|  | Reverse | GCGTGCATCCGCTTGTG |  |  |  |
| Cyc1 | Forward | GCTACCCATGGTCTCATCGT | NM_025567 | 207bp | 60℃ |
|  | Reverse | CATCATCATTAGGGCCATCC |  |  |  |
| COXII | Forward | CCATCCCAGGCCGACTAA | NC_025934 | 77bp | 60℃ |
|  | Reverse | AATTTCAGAGCATTGGCCATAGA |  |  |  |
